# Rapid molecular epidemiology investigations into two recent measles outbreaks in Israel detected from October 2023 to January 2024

**DOI:** 10.2807/1560-7917.ES.2024.29.16.2400202

**Published:** 2024-04-18

**Authors:** Efrat Bucris, Victoria Indenbaum, Tal Levin, Yara Kanaaneh, Keren Friedman, Tatyana Kushnir, Rivka Sheffer, Michal Savion, Matanelle Salama, Noa Di-castro, Kozita Labay, Maya Butera, Baraah Shihada, Zohar Mor, Yaniv Lustig, Neta S. Zuckerman

**Affiliations:** 1 Central Virology Laboratory, Public Health Services, Ministry of Health and Sheba Medical Center, Ramat-Gan, Israel; 2 Tel Aviv District Health Office, Ministry of Health, Tel Aviv, Israel; 3 District Health Office, Ministry of Health, Tiberias, Israel; 4 Division of Epidemiology, Ministry of Health, Jerusalem, Israel; 5 School of Health Sciences, Ashkelon Academic College, Ashkelon, Israel; 6 Faculty of Medicine, Department of Epidemiology and Preventive Medicine, Tel-Aviv University, Tel Aviv, Israel; * These authors contributed equally to the manuscript

**Keywords:** Measles, Molecular epidemiology, Whole Genome Sequencing (WGS)

## Abstract

Between late 2023 and early 2024, two measles outbreaks occurred in Israel, each caused by importation of measles virus strains of respective B3 and D8 genotypes. In this study, we validate transmission pathways uncovered by epidemiological investigations using a rapid molecular approach, based on complete measles virus genomes. The presented findings support this rapid molecular approach in complementing conventional contact tracing and highlight its potential for informing public health interventions.

Measles is a highly infectious disease that may lead to severe complications and can be prevented by vaccination. Despite Israel's high measles vaccination coverage (ca 94% as of 2023, according to Israel ministry of health), which mitigates the risk of extensive outbreaks, a recent surge of measles outbreaks globally has been accompanied by a considerable number of imported cases in the country, with, as witnessed in 2018–19, a potential for localised outbreaks [1]. We describe two recent unrelated measles outbreaks that occurred in two distant districts in Israel within a short timeframe, which were epidemiologically investigated with the support of a rapid molecular approach.

## Description of the outbreaks and laboratory diagnosis of cases

The first outbreak, with a total of 10 (4 female and 6 male) cases, all confirmed [2], was detected in October 2023, and determined to have occurred in the Tel-Aviv district between September and October 2023 (TLV outbreak). The outbreak involved two vaccinated adults (aged between 32 and 41 years) and eight children (< 4 years-old), with seven children who had received one dose of the measles, mumps, rubella and varicella (MMRV) vaccine, and one (index case) who was unvaccinated. The second outbreak, with a total of nine confirmed cases (4 female and 5 male), took place in the Tiberias district (TBS outbreak). It was detected in December 2023 and continued until January 2024, involving three adults aged between 19 and 70 years and six children (<8 years-old). Only one of nine cases in the TBS outbreak was vaccinated with at least one dose of MMRV vaccine. One case was considered to be naturally immunised.

Based on the epidemiological investigations [1], the TLV outbreak originated from a kindergarten with the source traced back to an importation from Moscow, Russia. Most likely, given their symptom onset 7 to 10 days following the index case, who showed symptoms in mid-September 2023, five cases from the same kindergarten were infected by the index case. The TLV1–3 cases were molecularly confirmed whereas two additional cases were confirmed via serology and were epidemiologically linked. Since a fourth case (TLV4) had attended the kindergarten, between 8 and 11 days after the previous cases, it is unclear whether TLV4 was directly infected by the index case or by any of the TLV1–3 cases. Case TLV7, a relative of TLV4, became infected through direct contact with TLV4. TLV5 was infected from TLV3 at a clinic facility. TLV6 was infected by another child in the same kindergarten who likely acquired measles from the index case. The transmission event was traced back to a visit to a museum (Figure A).

**Figure:**
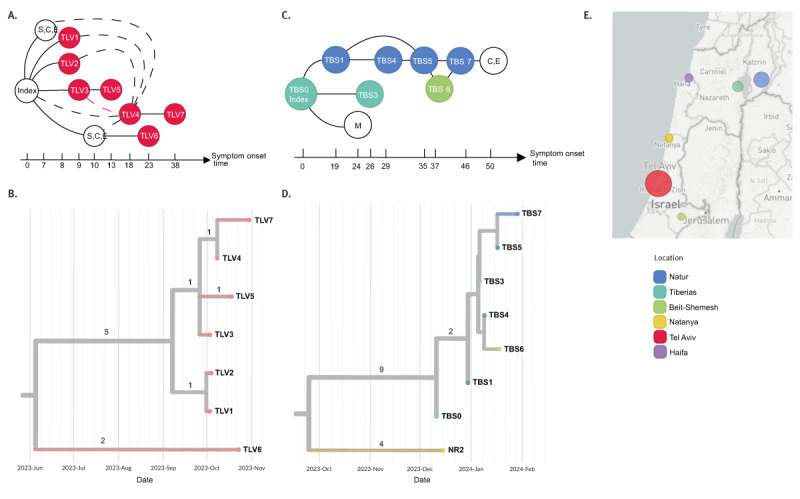
Transmission chains identified in two measles outbreaks, and phylogenetic analysis of genetic sequences of viral strains involved, Israel, October 2023–January 2024

C: clinically diagnosed; E: epidemiologically linked; M: molecularly confirmed; MeV: measles virus; S: serologically diagnosed.

A. Diagram representing transmission chains found through the epidemiological investigation that occurred during the measles outbreak in Tel-Aviv, which was caused by a MeV strain of genotype B3. Dashed lines denote potential transmission pathways for TLV4; the dashed red line denotes the inferred transmission route for TLV4 according to the phylogenetic analysis.

B. Phylogenetic analysis of MeV complete genome sequences from the Tel Aviv outbreak caused by a MeV strain of genotype B3. The number of MeV mutations are indicated on the branches.

C. Diagram representing transmission chains found through the epidemiological investigation that occurred during the measles outbreak in Tiberias district, which was caused by a MeV strain of genotype D8.

D. Phylogenetic analysis of MeV complete genome sequences from the outbreak in Tiberias district caused by a MeV strain of genotype D8 along with the sequence derived from an additional case with MeV genotype D8 unrelated to the outbreak, but from the same period (NR2). The number of MeV mutations are indicated on the branches.

E. Map of Israel indicating the location of the B3 and D8 sequenced cases.

In (A), (C) and (E), coloured circles represent the location of sequenced samples and white circles represent samples in the transmission chain which were not sequenced. In (E) some of the coloured circles represent the location of sequenced samples from individuals who were not involved in outbreaks in Israel. In (A) and (C), onset time is calculated, for each outbreak, as day of onset of symptoms, starting with the index cases (time = 0). In (B) and (D), coloured branches are a feature of the tree-drawing software, which colours branches directly leading to a leaf in the same colour as the leaf. Branches leading to nodes are not coloured.

The TBS outbreak was detected 3 months after the TLV outbreak. The epidemiological investigation concluded that it began with the importation of the virus from Azerbaijan by the index case, TBS0, who resides in Tiberias. Case TBS1, residing in the nearby village of Natur, TBS3, residing in Tiberias, and an additional individual from the nearby village of Maghar, contracted the virus from TBS0, where transmission took place in the emergency room of a local hospital. The outbreak persisted in Natur, where TBS4 contracted the virus from TBS1, a family member. TBS5 acquired the infection from TBS1 and TBS4 in a kindergarten and transmitted it to a family member, TBS7, who infected an additional family member (clinically and epidemiologically linked to laboratory confirmed case). TBS6 from Beit-Shemesh acquired the virus after spending time at TBS5 and TBS7 home in Natur (Figure C).

Seven of 10 and eight of nine samples from the TLV and TBS outbreaks, respectively, were diagnosed within 24–48 hours of symptoms’ onset using real-time PCR from urine or nasopharyngeal swab samples according to the World Health Organization (WHO) protocol [3]. Two of 10 from the TLV outbreak were diagnosed based on serology. One of 10 and one of nine from the TLV and TBS outbreaks, respectively, were diagnosed through epidemiological and clinical criteria.

Using conventional N450 fragment Sanger sequencing, the TLV outbreak was identified as associated with the B3 genotype, whereas the TBS outbreak was determined to be linked to the D8 genotype (data not shown). To determine the outbreak named strains, which are specific global measles virus (MeV) well-characterised variants known to have resulted in a widespread outbreak, we used the WHO global measles nt sequence database – MeaNS2 [4]. The named strain associated with the B3 TLV outbreak is MVs/Minnesota.US/15.17 (99% identity), whereas the named strain associated with the D8 TBS outbreak cases is MVs/Rudaki.TJK/49.21 (99.8% identity).

During the period covering the two outbreaks, two additional measles importations into Israel were recorded. The first importation was through case NR1, detected in the Haifa district, who had an infection with a B3 genotype MeV. NR1 was imported from Uzbekistan 3 months following the TLV outbreak, so was considered not related to this outbreak. This case was associated with the named strain MVs/Quetta.PAK/44.20 (100% identity). The second importation was through case NR2, detected in the Netanya district, who was imported from Kazakhstan at the time of the TBS outbreak and was thus determined as not related to this outbreak. NR2 was associated with the MVs/Rudaki.TJK/49.21 named strain, as were the strains identified in TBS outbreak samples, suggesting a common origin of the virus for both cases (NR2 from Kazakhstan and TBS0 from Azerbaijan, Table), which is further supported by close geographical proximity of those countries.

**Table:** Characteristics of measles cases for whom measles virus genetic sequences were obtained in the study, Israel, September 2023–January 2024 (n = 16 cases)

Case name	Onset time	Sample type	Cq	Vaccine doses	Named strain	WHO strain name	Genotype	Sequencing coverage	Average depth
TLV1	8	Urine	28.86	1	MVs/Minnesota.US/15.17	MVs/TelAviv.ISR/40.23	B3	100	6,627
TLV2	8	Urine	30.26	1	MVs/Minnesota.US/15.17	MVs/TelAviv.ISR/39.23	B3	100	6,129
TLV3	9	Urine	29.03	1	MVs/Minnesota.US/15.17	MVs/TelAviv.ISR/40.23/2	B3	100	6,810
TLV4	18	Urine	24.79	1	MVs/Minnesota.US/15.17	MVs/TelAviv.ISR/40.23/3	B3	100	7,399
TLV5	13	NPS	20	1	MVs/Minnesota.US/15.17	MVs/TelAviv.ISR/41.23	B3	100	7,412
TLV6	23	Urine	33.49	2	MVs/Minnesota.US/15.17	MVs/TelAviv.ISR/42.23	B3	91.4	744
TLV7	38	NPS	27.5	2	MVs/Minnesota.US/15.17	MVs/TelAviv.ISR/44.23	B3	100	5,684
NR1	125	NPS	14.27	0	MVs/Quetta.PAK/44.20	MVs/Haifa.ISR/04.24	B3	99.9	6,177
TBS0	0	NPS	25.2	0	MVs/Rudaki.TJK/49.21	MVs/Tiberias.ISR/50.23	D8	100	5,878
TBS1	19	Urine	17.16	0	MVs/Rudaki.TJK/49.21	MVs/Tiberias.ISR/52.23	D8	100	5,262
TBS3	26	NPS	17.36	NI	MVs/Rudaki.TJK/49.21	MVs/Tiberias.ISR/01.24/2	D8	100	6,962
TBS4	29	Urine	28.99	0	MVs/Rudaki.TJK/49.21	MVs/Tiberias.ISR/01.24	D8	100	5,212
TBS5	35	Urine	17.22	0	MVs/Rudaki.TJK/49.21	MVs/Tiberias.ISR/03.24	D8	100	5,634
TBS6	37	NPS	22.51	2	MVs/Rudaki.TJK/49.21	MVs/Tiberias.ISR/03.24/2	D8	100	4,910
TBS7	46	Urine	21.31	0	MVs/Rudaki.TJK/49.21	MVs/Tiberias.ISR/04.24	D8	100	5,162
NR2	15	Serum	28.3	0	MVs/Rudaki.TJK/49.21	MVs/Netanya.ISR/50.23	D8	100	6,297

Cq: quantification cycle in real-time PCR; NI: naturally immunised; NPS: nasopharyngeal swab; WHO: World Health Organization. Onset time is calculated, for each outbreak, as day of onset of symptoms, starting with the index cases (time = 0).

## Results of the molecular investigations

As part of routine epidemiological investigations conducted by the health department, rapid sequencing assays were employed to assist with the investigation of transmission chains for each outbreak. The rapid assay is based on MeV whole genome sequencing from the clinical samples, followed by phylogenetic analyses to assess transmission chains [5,6].

Whole genome sequencing was performed for seven B3 and eight D8 genotype samples (but for one, sequencing was unsuccessful), all of which were shown by investigations to be associated with the TLV and TBS outbreaks, respectively, as well as for two samples of individuals deemed unrelated by epidemiological inquiries, NR1 and NR2 (Table). Nucleic acids were extracted from these MeV-positive samples, MeV RNA was reversed transcribed and amplified via single one-step PCR reaction using specific measles whole genome primers [5], which we further enhanced with additional primers for the non-coding region between the matrix and fusion genes (MF-NCR) [7]. DNA libraries were prepared using Nextera-XT (Illumina) and sequencing was carried out using Ilumina MiSeq (Illumina) in under 48 hours from real-time PCR diagnosis of suspected samples. Samples were mapped to the B3 (GenBank accession number: KT732215.1) or D8 (GenBank accession number: KT732261.1) genotype sequences, based on the genotyping determined by the N450 Sanger sequencing. All samples but one achieved complete or near-complete coverage (Table). Phylogenetic trees were constructed using Nextrain Augur pipeline under the generalised time reversible (GTR) substitution model and visualised with Auspice [8].

The results of the phylogenetic analysis of the TLV outbreak (Figure B and E) overall correspond with those of the epidemiological investigation (Figure), revealing genomic connections among the affected children in the kindergarten. These included the TLV1–3 connections with the index case (for whom sequencing of a sample did not occur, as diagnosis was performed retrospectively). Moreover, according to this analysis, TLV3 transmitted the infection to TLV5, and TLV4 transmitted it to TLV7. Additionally, TLV6, not related to the kindergarten children, is linked to the outbreak through a shared branch in the tree leading back to one of the children in the kindergarten who was confirmed via serology and epidemiological linked to the outbreak and thus sequencing was not performed. TLV4 is positioned within a cluster sharing a single mutation, along with TLV3, which is separate from TLV1/2. Hence, it appears that TLV4 acquired the infection from TLV3 rather than the other confirmed cases in the kindergarten (Figure A and B).

NR1 was excluded from the analysis as it was not considered to be linked to the TLV outbreak due to the substantial time gap in its identification.

The phylogenetic analysis of the TBS outbreak (Figures D and E) corroborates the epidemiological narrative as well, but for one case, sequencing was unsuccessful. NR2, identified at the same timeframe as the TBS outbreak, is demonstrated by the phylogenetic analysis as not associated with the TBS outbreak. Although, the shared origin of NR2 and the TBS outbreak sequences, along with the relatively small difference in mutation counts, suggests a potential common origin of the virus strain.

## Discussion

In the current study, we describe independent measles importations to Israel as well as two outbreaks caused by two of these. The countries from which measles cases were imported into Israel (Azerbaijan, Kazakhstan, Russia) were recently listed among the 10 countries with the highest measles incidence rates, according to the United States Centers for Disease Control and Prevention (CDC) [9]. This, coupled with a marginal decline in vaccination coverage noted in Israel during the post-COVID-19 pandemic may heighten the risk of viral transmission and spread (Israel Ministry of Health, data not shown). Since measles was declared eliminated from Israel in 2015, the routinely-conducted comprehensive questionnaire-based epidemiological investigation following each confirmed case is imperative. This process is essential for severing transmission chains, identifying contacts, recommending active MMR vaccine or passive immunisation (immunoglobulins infusion) and implementing timely prevention measures, such as vaccination campaigns for affected communities. Nevertheless, these contact investigations are often incomplete due to lack of information resulting from limited cooperation, recall bias, time constraints or language barriers. Additionally, late notification reduces the time available for administering post-exposure prophylaxis (PEP). As a result, prevention measures are delayed, which may lead to the ongoing spread of the virus. The approach outlined and exemplified in this study, whole-genome sequencing of MeV directly from clinical samples followed by a high-resolution phylogenetic analysis, enables genome-based epidemiological investigations that enhance standard investigations and aid in breaking transmission chains. As demonstrated by the two recent B3 and D8 genotype measles outbreaks in Israel shown in this study, this approach generates complete or near-complete MeV genomes within a wide range of measles RNA concentrations in clinical samples (Cq values), it offers rapid and efficient processing from sample to sequence and complements ongoing investigations by providing molecular confirmation and clarification to epidemiological data received by health departments. Indeed, the phylogenetic analyses of both the TLV and TBS outbreaks reinforced the epidemiological investigations, helped to differentiate between samples which are related and unrelated to the outbreaks. Furthermore, they provided additional insights into the TLV4 infection route during the TLV outbreak, showcasing the effectiveness of this approach in instances where traditional investigations encounter difficulties in establishing clear connections between cases or are presented with inaccurate information.

In both outbreaks, some of the cases were only confirmed by serology and/or were clinically and epidemiologically linked, in addition to a sample for which sequencing could not be achieved, and thus could not be part of the molecular analysis. In cases when conventional methods reach an impasse, the lack of molecular information can limit the method described herein.

## Conclusion

The molecular approach and analyses for investigating measles outbreaks that are outlined herein could play a significant role in containing such outbreaks. If this approach is used alongside routine epidemiological investigations conducted by health districts and promptly implemented following case confirmation, it may have a substantial impact on public health, including maintaining Israel's measles elimination status.

## ^†^Authors’ correction

Upon publication of the article, the names of one author, Rivka Sheffer, were incorrectly spelled. The spelling was corrected on 19 April 2024 at the request of the corresponding author.
