# Authors’ correction for Euro Surveill. 2024;29(16)

**DOI:** 10.2807/1560-7917.ES.2024.29.17.240425c

**Published:** 2024-04-25

**Authors:** Eurosurveillance editorial team

**Affiliations:** 1 European Centre for Disease Prevention and Control (ECDC), Stockholm, Sweden

In the article entitled ‘Rapid molecular epidemiology investigations into two recent measles outbreaks in Israel detected from October 2023 to January 2024’ by Bucris et al., published on 18 April 2024, the names of one author, Rivka Sheffer, were incorrectly spelled. The spelling was corrected on 19 April 2024 at the request of the corresponding author.

